# Conservative management of postoperative incomplete lung torsion without reoperation: first case report with 2-year favorable outcomes

**DOI:** 10.1186/s12893-025-03427-1

**Published:** 2025-12-20

**Authors:** Keyao Dai, Yuan Zhang, Yudong Zhang, Yucong Deng, Shen Lao, Wei Wang

**Affiliations:** Department of Thoracic Surgery and Oncology, The Key Laboratory of Advanced Interdisciplinary Studies, National Center for Respiratory Medicine, National Clinical Research Center for Respiratory Disease, State Key Laboratory of Respiratory Disease, Guangzhou Institute of Respiratory Health, The First Affiliated Hospital, Guangzhou Medical University, 151 Yanjiang Road, Guangzhou, 510120 China

**Keywords:** Lung torsion, Postoperative complications, Conservative management, Case report

## Abstract

**Background:**

Lung torsion is a rare but serious postoperative complication in thoracic surgery, with traditional management requiring surgical intervention such as video-assisted thoracoscopic surgery or open thoracotomy. Reoperation carries significantly higher mortality rates and prolonged hospitalization compared to routine surgical outcomes. To date, no cases of successful conservative management of postoperative lung torsion have been reported in the literature. This case report presents the first successful conservative management of postoperative lung torsion, challenging the established surgical paradigm and offering new therapeutic possibilities.

**Case presentation:**

A 52-year-old male patient underwent dual-port video-assisted thoracoscopic surgery with right upper lobectomy and right lower lobe superior segmentectomy (RS6) for invasive pulmonary adenocarcinoma. Two hours after chest tube removal on postoperative day 2, the patient developed acute onset of profuse sweating, chest tightness, and dyspnea. Emergency chest radiography revealed complete lung atelectasis, and subsequent multidetector CT imaging combined with flexible bronchoscopy confirmed incomplete torsion of the right middle and lower lobes. A novel multimodal conservative approach was initiated, consisting of selective intrabronchial air insufflation every 48 h, noninvasive positive pressure ventilation (BiPAP), and maintained closed thoracic drainage. On postoperative day 7, three-dimensional imaging demonstrated significant resolution of atelectasis, and the chest tube was successfully removed. Three-month postoperative imaging revealed complete re-expansion of the residual right lung, with sustained expansion confirmed at 2-year follow-up without long-term sequelae.

**Conclusions:**

This case represents the first successful conservative management of postoperative lung torsion using multimodal conservative therapy with excellent long-term outcomes. Conservative management may be considered for carefully selected patients with early-diagnosed incomplete lung torsion who remain hemodynamically stable without tissue infarction. This approach requires intensive monitoring and immediate surgical backup availability. While offering a potential alternative to high-risk reoperation, surgical intervention remains the standard of care, and larger studies are needed to validate this approach and establish treatment protocols.

**Supplementary Information:**

The online version contains supplementary material available at 10.1186/s12893-025-03427-1.

## Introduction

Lung torsion is a rare but serious complication in thoracic surgery [1–3], most commonly manifesting as right middle lobe torsion following right upper lobectomy [4–8]. Traditionally, surgical intervention has been considered the only definitive treatment, including video-assisted thoracoscopic surgery (VATS) or open thoracotomy with resection or repositioning of the twisted lobe (Supplementary Table 1) [4, 5, 7, 9–21]. However, patients undergoing reoperation face significantly higher in-hospital mortality rates and prolonged hospitalization, with reoperation mortality reaching 5.13% compared to routine surgical outcomes [22]. To our knowledge, no cases of successful conservative management of postoperative lung torsion have been previously reported in the literature. We present the first case of residual lung torsion following right upper lobectomy and right lower lobe superior segmentectomy (RS6), where the twisted lung was successfully repositioned through innovative multimodal conservative management, restoring pulmonary function with excellent 2-year follow-up outcomes. This breakthrough challenges the traditional surgical paradigm and offers new therapeutic possibilities for selected patients. This study was approved by the institutional ethics committee (approval number ES-2025-K189-01).

## Case presentation

### Initial presentation and surgery

**Fig. 1:**
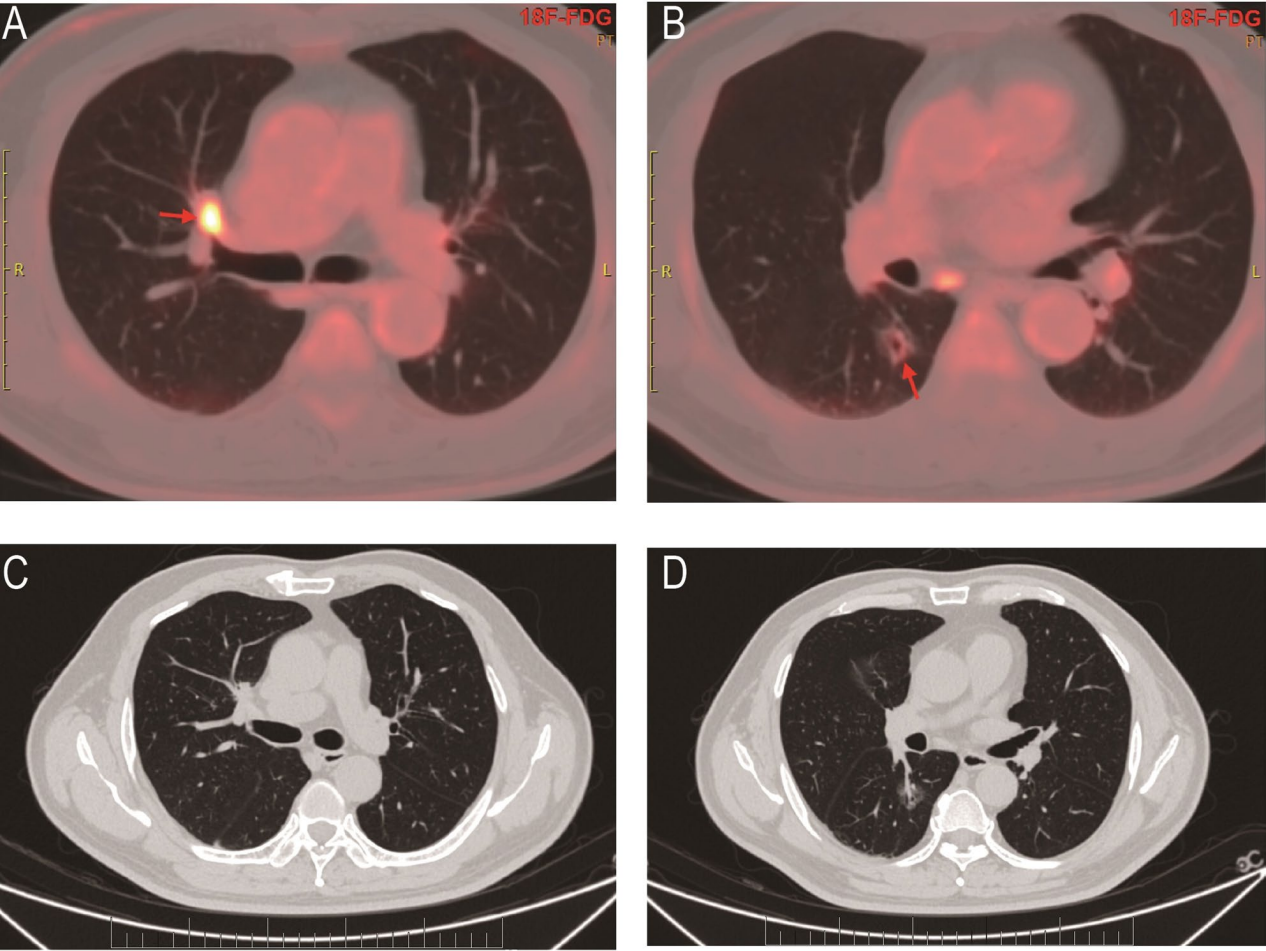
Preoperative PET-CT shows synchronous primary lung cancers. (**A**, **C**) A hypermetabolic tumor in the RS3 segment of the right upper lobe. (**B**, **D**) A separate primary tumor presenting as mixed-density nodules with mild hypermetabolism in the RS6 segment of the right lower lobe

**Fig. 2:**
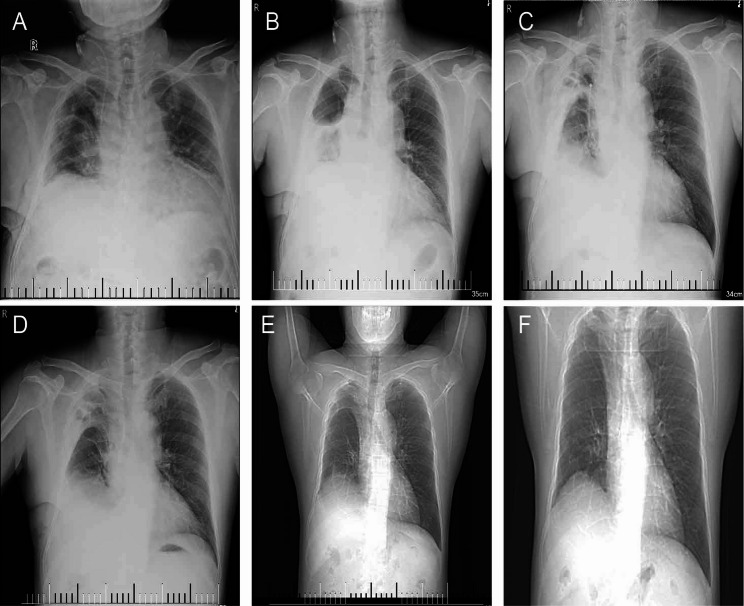
Serial imaging of the clinical course. (**A-D**) Chest radiographs. (**A**) Postoperative day 2, showing satisfactory lung re-expansion. (**B**) Postoperative day 3, showing complete atelectasis 2 h after chest tube removal. (**C**) After 3 days of conservative treatment, showing radiographic improvement with partial re-expansion. (**D**) At discharge. (**E-F**) CT topograms (scout views). (**E**) Three months postoperatively, showing complete re-expansion of the residual right lung. (**F**) Two years postoperatively, demonstrating sustained lung expansion without recurrence

A 52-year-old male patient underwent PET-CT examination, which revealed two separate hypermetabolic nodules suggestive of synchronous primary malignancies: one located in the anterior segment of the right upper lobe (RS3, near the mediastinal pleura), and the other in the RS6 segment (Fig. 1). At our institution, the patient underwent dual-port video-assisted thoracoscopic surgery (VATS) with right upper lobectomy, RS6 segmentectomy, systematic mediastinal lymph node dissection, and adhesiolysis. Histopathological examination confirmed invasive pulmonary adenocarcinoma. Postoperative day 2 chest radiography demonstrated satisfactory lung re-expansion (Fig. 2A), prompting chest tube removal.

### Acute complication and diagnosis

**Fig. 3:**
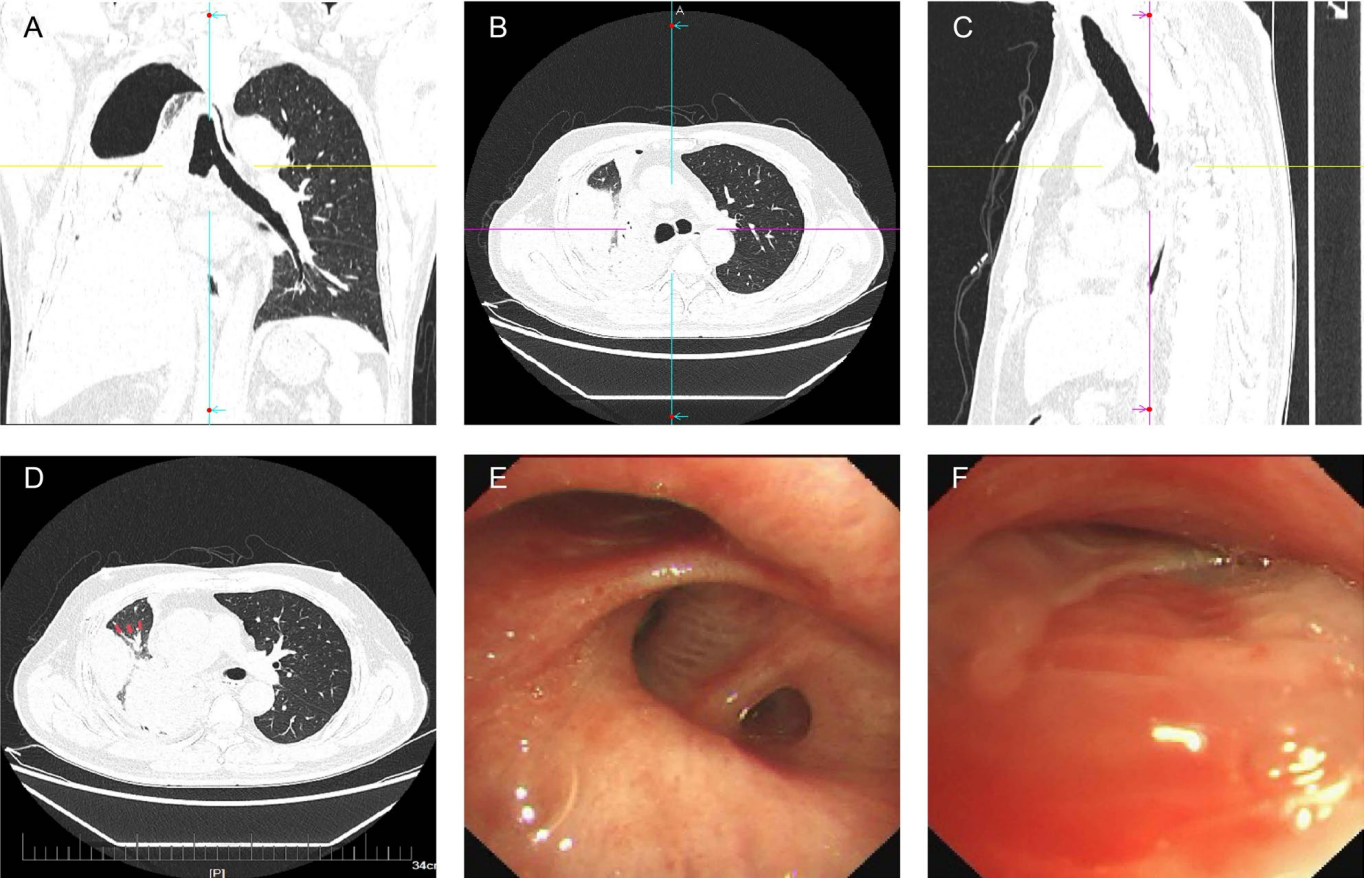
Coronal (**A**), axial (**B**), and sagittal (**C**) CT images demonstrating distal bronchial obstruction and atelectasis of the right lung. (**D**) Axial CT image revealing the characteristic “antler sign” of lung torsion—abnormal bronchial orientation with acute angulation and rotation, considered pathognomonic for lung torsion [25]. Notably, despite the presence of torsion, maintained blood flow is observed, consistent with the imaging features of incomplete torsion. (**E**) Bronchoscopic view showing torsion and stenosis of the right middle and lower lobe bronchi. (**F**) The “fish-mouth sign” of the middle lobe bronchus—slit-like bronchial opening resulting from 180°axial rotation, confirming the diagnosis of torsion [26]

Two hours following chest tube removal, the patient developed acute onset of profuse sweating, chest tightness, and dyspnea in the absence of fever or pleuritic chest pain. Emergency chest radiography revealed complete lung atelectasis (Fig. 2B). Closed thoracic drainage was performed emergently, yielding 200 mL of serous fluid, raising suspicion for lung torsion. Subsequent multidetector CT imaging in three orthogonal planes (coronal, sagittal, and axial) combined with flexible bronchoscopy confirmed incomplete torsion of the right middle and lower lobes (Fig. 3A-F).

### Conservative management protocol

Following confirmation of diagnosis, we initiated a multimodal conservative approach consisting of three components:

1. Selective intrabronchial air insufflation: Performed every 48 h targeting the twisted bronchi (detailed procedural parameters demonstrated in Video 1) to re-establish patency of the obstructed airways [23].
2. Noninvasive positive pressure ventilation: BiPAP spontaneous mode [inspiratory positive airway pressure (IPAP)/expiratory positive airway pressure (EPAP): 12/4 cmH2O] administered three times daily for 2-hour sessions, supplemented with continuous nasal cannula oxygen therapy (3 L/min) between treatments [24].
3. Closed thoracic drainage: Maintained to reduce pleural cavity pressure and monitor for complications.

Broad-spectrum antibiotic prophylaxis was instituted with systematic radiological monitoring. Given the absence of clinical deterioration or radiographic evidence of pulmonary infarction (absence of fever or pleuritic pain), and serial chest radiographs demonstrating progressive treatment response (Fig. 2C), operative intervention was deferred following comprehensive informed consent discussions regarding risks and benefits of both surgical and conservative approaches.

### Clinical outcome

**Fig. 4:**
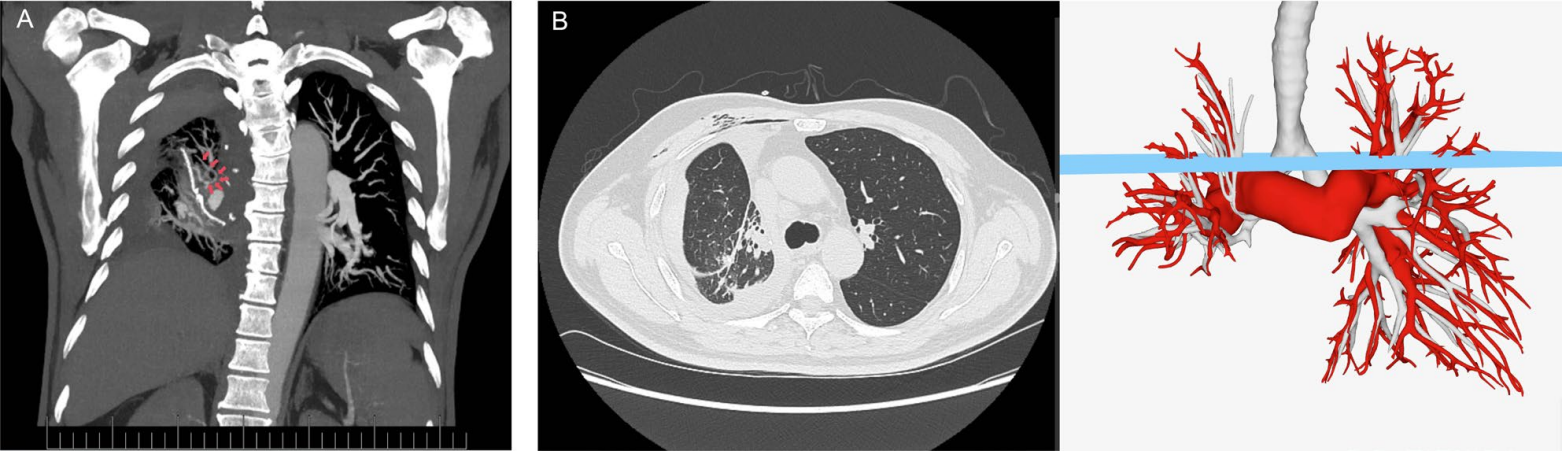
CT imaging and 3D reconstruction of pulmonary vasculature and airways after 3 days of conservative treatment. (**A**) Mild residual vascular swirling in the distal right lung vasculature (arrow). (**B**) Despite these findings, axial CT at the previously obstructed level and corresponding 3D reconstruction model demonstrate good distal opacification with significant resolution of atelectasis

On postoperative day 7, corresponding to the third day of conservative treatment, three-dimensional imaging showed significant atelectasis resolution. Volume-rendered reconstruction of the pulmonary arteries, veins, and airways confirmed improved lung expansion (Fig. 4). Given this radiographic improvement, the chest tube was successfully removed. As a precautionary measure, the patient remained hospitalized for an additional 6 days of observation. The patient’s clinical course remained uncomplicated, and discharge was authorized following confirmatory chest radiography (Fig. 2D).

Three-month postoperative CT topogram revealed complete re-expansion of the residual right lung with satisfactory filling of the entire thoracic cavity (Fig. 2E). At 2-year follow-up, topographic imaging continued to demonstrate sustained expansion without apparent long-term sequelae (Fig. 2F).

## Discussion

This represents the first successful conservative management of postoperative lung torsion, fundamentally challenging the established treatment paradigm that has mandated surgical intervention for over five decades. While traditional management strategies include direct resection (54.4%), repositioning followed by immediate resection (12.6%), or repositioning alone (33%) [10], our case demonstrates that conservative treatment can achieve complete resolution with superior outcomes compared to reoperation risks.

Our multimodal conservative approach had three synergistic components. Each component specifically addressed the pathophysiology of lung torsion. Closed thoracic drainage reduced intrapleural pressure, while selective intrabronchial air insufflation increased endobronchial pressure, creating a favorable pressure gradient for spontaneous detorsion [23]. Notably, SII represents a key innovation of “repurposing an established technique for a novel indication”—having been successfully applied to atelectasis, we adapted it for incomplete lung torsion. The application logic is based on a fundamental mechanistic analogy: both atelectasis and incomplete lung torsion share a common pathophysiologic endpoint—bronchial luminal collapse due to external mechanical forces (whether from mucus plug compression or physical torsion). SII addresses this by directly injecting air into the target bronchi, using pneumatic pressure to mechanically splint the collapsed lumen—analogous to reopening a compressed water pipe. Therefore, we transferred this technique, proven effective for “mechanical obstruction,” to address “torsional angulation”—a unique form of mechanical obstruction. Concurrent noninvasive ventilation maintained positive end-expiratory pressure, stabilizing the airways and preventing re-torsion. While we utilized a simple syringe-based delivery system rather than sophisticated pressure-monitoring equipment, this bedside technique using readily available instruments offers greater practical applicability for this rare complication, facilitating adoption in community hospital settings without requiring specialized equipment. This physiologically rational strategy addressed both mechanical obstruction and hemodynamic consequences without surgical trauma, representing a paradigm shift in therapeutic approach.

Several key factors contributed to our successful outcome and may serve as selection criteria for future cases. Early recognition within hours of symptom onset prevented irreversible ischemic changes. The incomplete nature of torsion with preserved partial vascular flow on imaging was crucial, distinguishing this case from complete torsions. The patient’s hemodynamic stability and absence of tissue infarction markers—fever, pleuritic pain, or hemoptysis—supported our conservative strategy under intensive monitoring.

The clinical implications extend beyond this individual case. Given that reoperation is associated with significantly higher mortality rates and substantially prolonged hospitalization [22], our conservative approach offers a transformative alternative that spares appropriately selected patients from surgical risks while achieving equivalent or superior outcomes. The 2-year follow-up demonstrates not only immediate success but sustained long-term efficacy, validating this innovative therapeutic strategy.

While our approach requires careful patient selection, the success factors we identified provide a practical framework for clinical decision-making: hemodynamic stability, incomplete torsion with preserved vascular flow, early diagnosis before tissue infarction, and availability of intensive monitoring. These criteria, while requiring validation in larger cohorts, offer actionable guidance for clinicians facing similar cases (Supplementary Table 2).

## Conclusion

We report the first successful conservative management of postoperative lung torsion using multimodal conservative therapy with excellent 2-year outcomes. Conservative management may be considered for carefully selected patients with early-diagnosed incomplete lung torsion who remain hemodynamically stable without tissue infarction. This approach requires intensive monitoring and immediate surgical backup. While offering a potential alternative to high-risk reoperation, surgical intervention remains the standard of care. Larger studies are needed to validate this approach and establish treatment protocols.

## Supplementary Information

Supplementary Material 1. Demonstration of Selective Intrabronchial Air Insufflation (SII).

Supplementary Material 2.

Supplementary Material 3.

## Data Availability

No datasets were generated or analysed during the current study.
