# Supplementary material for: Conservative management of postoperative incomplete lung torsion without reoperation: first case report with 2-year favorable outcomes
Source: BMC Surg. 2025 Dec 20;26:70. doi: 10.1186/s12893-025-03427-1 (PMC12831388; doi:10.1186/s12893-025-03427-1)
Supplement: Supplementary file 2 — Supplementary Material 2. [file 12893_2025_3427_MOESM2_ESM.docx]

**Supplementary Table 1.** **Summary of Pulmonary Torsion Complications, Interventions, and Outcomes.**

| **Author** | **Publication Year** | **Number of Cases** | **Twisted Lung Lobe(s)** | **Torsion Type** | **Treatment** | **Outcome** |
| --- | --- | --- | --- | --- | --- | --- |
| E. H. Stratemeier, et al.[1] | 1954 | 1 | Left Lung | Complete | No surgical intervention for torsion | Death |
| Robert M. Shorr, et al.[2] | 1987 | 1 | Left Lung | Complete | Re-operation: Resection (Pneumonectomy) | Death |
| Efstratios Apostolakis, et al.[3] | 2006 | 1 | Left Upper Lobe | Complete | Re-operation: Resection (Pneumonectomy) | Death on postoperative day 26 (cerebral embolism after re-operation, died in ICU) |
| Charles C. Sticco, et al.[4] | 2007 | 1 | Right Middle Lobe | Complete | Re-operation: Resection (Right middle lobectomy) | Good recovery |
| Stefano Schena, et al.[5] | 2008 | 1 | Right Lower Lobe | Partial | Re-operation: Reduction via VATS | Discharged on postoperative day 7, good recovery |
| Simon Hennink, et al.[6] | 2008 | 1 | Left Upper Lobe | Complete | Re-operation: Resection (Thoracotomy, Pneumonectomy) | Discharged on day 57 (from initial surgery), good recovery |
| Takashi Eguchi, et al.[7] | 2008 | 1 | Left Upper Lobe | Complete | Re-operation: Resection (Thoracotomy, Left upper lobectomy) | Discharged 14 days after re-operation, good recovery |
| Chih-Hao Chen, et al.[8] | 2009 | 1 | Right Middle Lobe | Complete | Re-operation: Resection (Right middle lobectomy) | Hospitalized for 10 days, good recovery |
| Ho Kyung Sung, et al.[9] | 2012 | 1 | Right Middle Lobe | Complete | Re-operation: Resection (VATS Right middle lobectomy) | Discharged on postoperative day 12 (from initial surgery), good recovery |
| Masahiro Irie, et al.[10] | 2014 | 1 | Right Lung | Complete | Re-operation: Reduction via VATS | Discharged on postoperative day 6, good recovery; asymptomatic without recurrence at 5-month follow-up |
| Metesh Nalin Acharya, et al.[11] | 2015 | 1 | Right Middle Lobe | Complete | Re-operation: Resection (Uniportal VATS Right middle lobectomy) | Discharged on day 15 (from initial surgery), postoperative infection but good recovery |
| Mohan Venkatesh Pulle, et al.[12] | 2020 | 2 | Right Middle Lobe (Both cases) | Incomplete / Complete | Re-operation (Both VATS: 1 resection, 1 reduction) | Both recovered well |
| Britton B. Donato, et al.[13] | 2023 | 2 | Right Middle Lobe (Both cases) | Complete | Re-operation: Resection (Thoracotomy, Right middle lobectomy) | Case 1: Discharged on day 88 (from liver transplant), complicated course but good recovery. |
|  |  |  |  |  |  | Case 2: Discharged on day 43, with complications but eventual recovery. |
| Aurelie Janet-Vendroux, et al.[14] | 2023 | 3 | Right Middle Lobe | Incomplete (2 cases); Complete (1 case) | Re-operation (2 reductions, 1 right middle lobectomy) | All survived, no complications at 12-month follow-up |

**References**

1. Stratemeier EH, Barry JW. Torsion of the Lung Following Thoracic Trauma: A Case Report. Radiology. 1954;62:726–7. https://doi.org/10.1148/62.5.726.

2. Shorr RM, Rodriguez A. Spontaneous Pulmonary Torsion. Chest. 1987;91:927–8. https://doi.org/10.1378/chest.91.6.927.

3. Apostolakis E, Koletsis EN, Panagopoulos N, Prokakis C, Dougenis D. Fatal stroke after completion pneumonectomy for torsion of left upper lobe following left lower lobectomy. J Cardiothorac Surg. 2006;1:25. https://doi.org/10.1186/1749-8090-1-25.

4. Sticco CC, Andaz S, Fox S. Middle lobe torsion after right upper lobectomy: A report of video-assisted thoracoscopic management. The Journal of Thoracic and Cardiovascular Surgery. 2007;134:1090–1. https://doi.org/10.1016/j.jtcvs.2007.05.046.

5. Schena S, Veeramachaneni NK, Bhalla S, Gutierrez FR, Patterson GA, Kreisel D. Partial lobar torsion secondary to traumatic hemothorax. The Journal of Thoracic and Cardiovascular Surgery. 2008;135:208-209.e2. https://doi.org/10.1016/j.jtcvs.2007.08.053.

6. Hennink S, Wouters MWJM, Klomp HM, Baas P. Necrotizing pneumonitis caused by postoperative pulmonary torsion. Interactive CardioVascular and Thoracic Surgery. 2008;7:144–5. https://doi.org/10.1510/icvts.2007.158378.

7. Eguchi T, Kato K, Shiina T, Kondo R, Yoshida K, Amano J. Pulmonary torsion of the lingula following a segmentectomy of the left upper division. Gen Thorac Cardiovasc Surg. 2008;56:505–8. https://doi.org/10.1007/s11748-008-0281-4.

8. Chen C-H, Hung T-T, Chen T-Y, Liu H-C. Torsion of right middle lobe after a right upper lobectomy. J Cardiothorac Surg. 2009;4:16. https://doi.org/10.1186/1749-8090-4-16.

9. Sung HK, Kim HK, Choi YH. Re-thoracoscopic surgery for middle lobe torsion after right upper lobectomy. European Journal of Cardio-Thoracic Surgery. 2012;42:582–3. https://doi.org/10.1093/ejcts/ezs214.

10. Irie M, Okumura N, Nakano J, Fujiwara A, Noguchi M, Kayawake H, et al. Spontaneous Whole-Lung Torsion After Massive Pleural Effusion and Atelectasis. The Annals of Thoracic Surgery. 2014;97:329–32. https://doi.org/10.1016/j.athoracsur.2013.04.133.

11. Acharya MN, Haqzad YS, Rao JN, Socci L. Uniportal thoracoscopic management of middle lobe torsion after upper lobectomy. Asian Cardiovasc Thorac Ann. 2015;23:1129–31. https://doi.org/10.1177/0218492315592994.

12. Pulle M, Asaf B, Puri H, Kumar A. Successful video-assisted thoracoscopic management of the right middle lobe torsion: A rare complication of right upper lobectomy – A report of two cases. Lung India. 2020;37:530. https://doi.org/10.4103/lungindia.lungindia_254_19.

13. Donato BB, Sewell M, Al Harakeh H, Sen A, Patel BM, Morgan P, et al. Spontaneous middle lobe torsion: An institutional case series. JTCVS Techniques. 2023;20:176–81. https://doi.org/10.1016/j.xjtc.2023.04.006.

14. Janet-Vendroux A, Al Zreibi C, Reverdito G, Arame A, Badia A, Masmoudi H, et al. Middle lobe suffering due to malposition and 180° tilt of the 2 remaining lobes after right upper lobectomy. Interdisciplinary CardioVascular and Thoracic Surgery. 2023;36:ivad038. https://doi.org/10.1093/icvts/ivad038.
