# Supplementary material for: Conservative management of postoperative incomplete lung torsion without reoperation: first case report with 2-year favorable outcomes
Source: BMC Surg. 2025 Dec 20;26:70. doi: 10.1186/s12893-025-03427-1 (PMC12831388; doi:10.1186/s12893-025-03427-1)
Supplement: Supplementary file 3 — Supplementary Material 3. [file 12893_2025_3427_MOESM3_ESM.docx]

**Supplementary Table 2. Proposed Selection Criteria for Conservative Management of Postoperative Lung Torsion.**

| **Indications** | **Absolute Contraindications**: |
| --- | --- |
| - **Essential Criteria (all must be met): Early diagnosis (<6 hours), Incomplete torsion (partial vascular flow on CT), Hemodynamic stability, Absence of tissue infarction signs** - **Supportive Factors**: Availability of intensive monitoring, Immediate surgical backup, Patient understanding and consent | - Complete torsion - Hemodynamic instability - Evidence of pulmonary infarction - Inability to provide intensive monitoring |
